# Unraveling the pharmacological and therapeutic potential of Ranolazine beyond antianginal drug use: a new insight

**DOI:** 10.3389/ebm.2025.10604

**Published:** 2025-09-03

**Authors:** Dhirendra Singh, Joy Awulika Oladimeji-Salami, Abidemi James Akindele

**Affiliations:** ^1^ Department of Pharmacology, M.M College of Pharmacy, Maharishi Markandeshwar (Deemed to be University), Mullana, Ambala, Haryana, India; ^2^ Special Duties Department, National Biotechnology Development Agency, Abuja, Nigeria; ^3^ Department of Pharmacology, Therapeutics and Toxicology, Faculty of Basic Medical Sciences, College of Medicine, University of Lagos, Lagos, Nigeria

**Keywords:** anticancer, cardioprotective, renalprotective, neuroprotective, hepatoprotective

## Abstract

Ranolazine (RAN) is an acetanilide and piperazine derivative that selectively blocks the late sodium current in cardiac cells and is prescribed in adults as an add-on medication for the symptomatic management of patients with stable angina pectoris who are insufficiently managed or intolerant of first-line antianginal treatments. RAN was first approved by the U.S. Food and Drug Administration (FDA) in 2006 and the European Medicine Agency in 2008 for the treatment of chronic stable angina. RAN has no substantial effect on hemodynamic indicators, including heart rate and blood pressure. RAN also slows fatty acid oxidation, which increases glucose oxidation, lowers lactic acid generation, and optimizes heart performance. Besides its antianginal effect, RAN has recently revealed additional pharmacological properties such as neuroprotective, hepatoprotective, renal protective, cardioprotective, and antidiabetic effects and other beneficial pharmacological activities. We choose to write this current review paper to address the many hidden pharmacological and therapeutic potentials of RAN beyond its antianginal activity.

## Graphical Abstract

**Figure:**
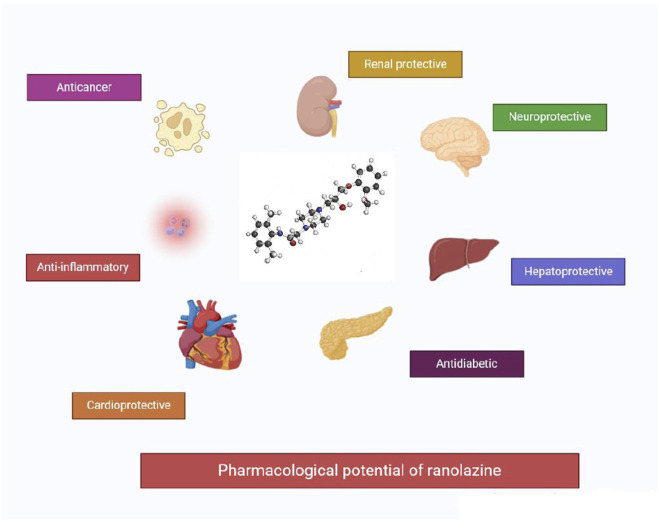
Pharmacological potential of ranolazine.

## Impact statement

Drug re-purposing, finding new therapeutic applications for old or existing drugs, provides the avenue to increase the therapeutic options for the treatment of disease conditions with the possible benefit of enhanced efficacy and safety profile. Beyond its antianginal action, Ranolazine exhibits a variety of pharmacological actions which can be explored for therapeutic benefits. This review extensively sheds light on a number of these pharmacological actions to broaden knowledge and spheres of potential therapeutic applications of Ranolazine.

## Introduction

**FIGURE 1:**
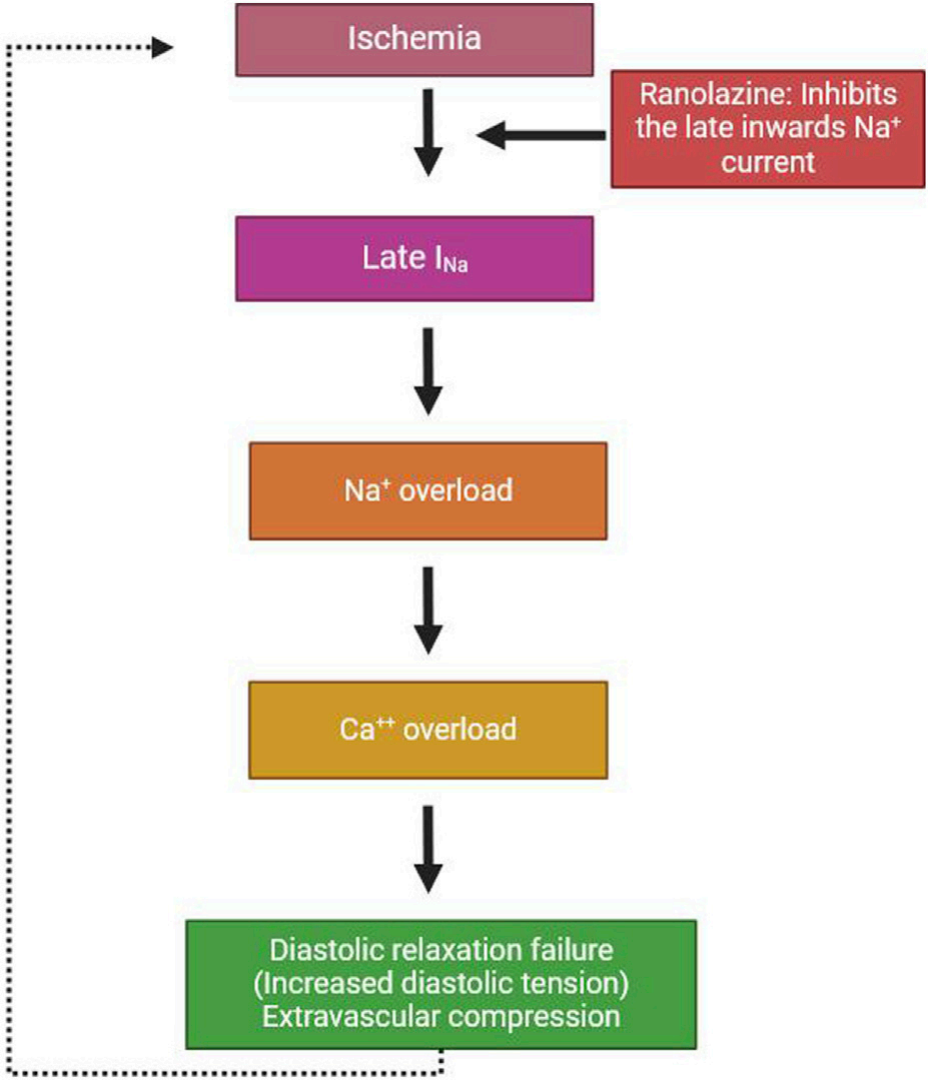
Mechanism of action of ranolazine.

Ranolazine (RAN) is N-(2, 6-dimethyl phenyl)-4(2-hydroxy-3-[2-methoxyphenoxy] - propyl)-1-piperazine acetamide dihydrochloride. It is an active piperazine whose anti-ischemic effect was originally attributed to the selective inhibition of fatty acid oxidation with a consequent shift of metabolism to more energy-efficient glucose oxidation [1]. An alternative mechanism of action proposed in the past for RAN was the inhibition of β_1_ and β_2_ adrenoceptors [2]. However, this mechanism (which is associated with sympathetic nervous system regulation of heart rate and contractility) is less prominent compared to RAN’s primary action on cardiac ion channels. It is a less significant involvement at the therapeutic concentration of RAN for the treatment of angina, with the main mechanism being linked to inhibition of the late sodium current in cardiac myocytes. This effect reduces intracellular calcium overload and improves myocardial relaxation and oxygen efficiency. At the clinical level, RAN decreases the current of sodium and potassium ion channels. It has been well studied that inhibition of the late phase of the inward sodium current occurs during cardiac repolarization [3]. In pathological conditions, a rise in calcium ion concentrations contributes to increased sodium-calcium interaction, which induces an increase in the cytosolic calcium concentration [4]. Calcium overload is thought to be the factor that induces reduced left ventricular relaxation during moderate ischemia as well as reperfusion. Increased left ventricular diastolic wall stress compromises myocardial tension circulation, which continues to rise still. Moreover, calcium overload has harmful impacts on myocardial electrical activity, predisposing to ventricular tachycardia [5]. Although this mechanism has been well studied mainly in rodents, the anti-ischemic activity of RAN due to late Na-channel suppression of myocardial perfusion lacks evidence to support this mechanism in patients with ischemic heart disorders. RAN slows the delayed rectifying K^+^ current at therapeutic doses and enhances the Q-T interval [6]. The total effect of RAN on the action potential period is equilibrium between the combined effects of rectifier potassium current as well as late sodium current suppression, which prolongs the QT interval by 2–6 ms [7]. Figure 1 shows the mechanism of action of RAN.

The pharmacologically induced attenuation of the late sodium current enhances cardiac diastolic relaxation by decreasing diastolic wall stress. This ultimately results in an improvement of segmental myocardial ischemia.

RAN was first used in therapeutic settings over 25 years ago. It is widely used to treat some disorders and is safe and effective in many cases. Many preclinical and clinical experiments show that RAN may exert cellular protective effects by specifically suppressing the late sodium inward current (late iNa). In the past few years, RAN has been associated with numerous positive properties, such as anticancer, renoprotective, hepatoprotective, neuroprotective, cardioprotective, analgesic, and anti-inflammatory activity, and other benefits independent of its antianginal function.

RAN modulates several cellular pathways like TNF-α, NF-κB, Capase-3, IL-1β, IL-6, PPAR-γ, Bax bcl-2, Notch2/Hes1, AKT-eNOS, COX-2, and ERK, which is activity independent of its cardiac protective mechanism.

## Search strategies

The literature search was done on multiple electronic databases. These include Web of Science, PubMed, Scopus, and Google Scholar. Appropriate search terms and combinations were used, including ranolazine, pharmacokinetics, neuroprotective, hepatoprotective, renoprotective, cardioprotective, and antidiabetic effects.

## Pharmacokinetics

RAN is available as an oral tablet for therapeutic use and as an intravenous formulation for experimental application. Initially, oral RAN was evaluated as an instant release (IR) formulation. RAN IR has an overall terminal removal half-life of 1.4–1.9 h and a 10-fold peak-trough gap of 240–400 mg three times per day [8]. RAN is now commonly available as a sustained-release (SR) formulation with a more extended absorption phase, with a maximal plasma concentration (Cmax) usually seen 4–6 h after oral administration and an estimated apparent total elimination half-life of 7 h after steady state. The peak-trough difference at 500–1000 mg twice/day is only 1.6-fold, which is much improved over that of the IR formulation [9–11]. The steady state is usually reached within 3 days of twice-daily dosing. RAN plasma amounts that are clinically beneficial for chronic angina range from 2 to 6 μmol/L [12, 13]. The oral bioavailability of RAN is 30%–55% and is not influenced by food. RAN is approximately 65% bound to serum protein, mainly α1-acid glycoprotein [14]. RAN is mainly cleared by the liver metabolic enzyme cytochrome P450 (CYP) 3A4 (70–85%) and is a substrate of P-glycoprotein. Additional processes include CYP2D6 metabolism (10–15 percent), glucuronidation (<5 percent), and renal excretion of unchanged RAN (<5 percent) [8].

## Anticancer effects

Driffort et al. found that RAN repressed the pro-invasive shape of human breast cancer MDA-MB-231 cells and decreased the localized extracellular matrix degradation activity [15]. Qiu et al. and Lee et al. validated similar findings and discovered that the anti-invasive action might occur independently of proliferation [16, 17]. Qiu and his group found that RAN’s anti-invasive activity was dose-related, with concentrations as low as 2.5 μM during hypoxia [16]. Guzel et al. found that, in human colorectal cancer cells, (i) hypoxia markedly increased Matrigel invasion and (ii) therapeutic dosages of RAN decreased invasiveness without compromising proliferative ability or cell survival [18].

Rizaner and colleagues demonstrated that for robust metastatic rat prostate cancer Mat-LyLu cells, RAN (i) hindered Matrigel migration under both normoxic and hypoxic circumstances and (ii) decreased the proportion of cells in the lung metastases showing Nav1.7 [19]. Pemmireddy and team examined the anticancer action of RAN on 1,2-Dimethyl hydrazine (DMH)-induced colon cancer in mice and found that RAZ substantially reduced colon cancer in mice, most likely because of cancer cell growth deregulations [20]. Using the Dunning model of rat prostate cancer, Bugan and coworkers demonstrated in double-blind tests that gavage administration of 2.5–5 μM RAN inhibited lung metastasis by as much as 63% [21]. Guth et al. demonstrated that RAN (i) inhibited tumor development and (ii) boosted anti-cancer immunity, as shown by reduced tumor CD8^+^ T-cells Tim3 content, enhanced macrophages, and lowered blood myeloid immunosuppressive monocytes in the TRAMPC1 genetic mice model of prostate cancer [22]. Lastly, Lasheras-Otero et al. demonstrated that RAN inhibited liver metastases in a mouse model of melanoma [23].

## Cardioprotective effects

Tocchetti et al. revealed that RAN could avert doxorubicin-induced cardiac failure in mice and HL-1 cardiomyocytes via lowering ROS production [24]. Furthermore, RAN has been shown to mitigate cardiac dysfunction induced by trastuzumab, which is believed to mediate its activity by inhibiting the generation of ROS [25]. De Lorenzo and teammates found that RAN mitigated not just the cardiotoxic adverse effects of trastuzumab but also of pertuzumab and trastuzumab-emtansine (TDM1) when employed in combinatorial therapies both *in vitro* and *in vivo* [26]. Cappetta et al. conducted an experiment using RAN and stated that it could protect cardiomyocytes from doxorubicin-caused oxidative damage [27]. RAN could attenuate MTX-caused oxidative damage in H9c2 cardiomyocytes by reducing MDA, LOOH, AOPPs, and XO activity, maintaining T-SH, CAT, and TAC levels, and prohibiting the HIF-1α inflammatory cascade [28]. Jiang et al. reported that therapy with RAN in Phospholamban (PLN) knockout hiPSCs-CMs could significantly repair Ca^2+^ handling abnormalities and cellular energy metabolism, thus alleviating the PLN knockout phenotype of HF [29].

In high glucose-treated cardiac fibroblasts, RAN decreased pyroptosis, prevented collagen deposition, and enhanced heart function via enhancing miR-135b expression [30]. Furthermore, RAN protected against diabetic cardiomyopathy-induced apoptosis in rats via activation of the NOTCH1/NRG1 signaling cascade [31]. Tawfik and team showed that RAN administration ameliorated the isoprenaline-mediated myocardial damage in both nondiabetic and diabetic rats by improving histopathological scores, reducing apoptotic markers, and modulating AMPK activity [32]. Le DE and his team proved that RAN increased both resting and stress-induced cardiac adenosine levels and caused small-vessel vasodilation, which improved ischemia in dogs [33]. RAN also showed a positive effect on cardiomyocytes subjected to ischemia/reperfusion, but only when used during ischemia, and this effect is accomplished through improving calcium regulation during ischemia [34].

Tantray et al confirmed that RAN had a protective role in myocardial infarction, similar to ischemic preconditioning facilitators, via promoting myocardial Nitric oxide, Adenosine, Bradykinin, and K^+^ATPase levels in an isolated heart [35]. In anaesthetized rabbits subjected to ischemia and reperfusion, RAN lowered infarct size and raised salvage area index, activating a process similar to PreC and PostC that required activation of the RISK axis [36]. Feng and co-workers demonstrated that chronic RAN treatment effectively reduced the increased concentrations of NE and BNP-45 caused by CHF and improved LV function in CHF rats [37]. RAN increased cardiac function and decreased the level of heart injury in rats with congestive heart failure, which is likely due to the activation of AKT phosphorylation [38]. RAN attenuates pressure overload-mediated cardiac hypertrophy and systolic and diastolic activity by restoring Na^+^ and Ca^2+^ handling, preventing downstream hypertrophic pathways, and reducing ER stress [39].

In an animal model of heart failure, RAN ameliorated cardiac remodeling and improved systolic and diastolic performance by normalizing Ca^2+^ storage [40]. Coppini and colleagues showed that acute RAN treatment lowered intracellular Na^+^ and Ca^2+^ levels as well as CaMKII activity, which contributed to the decrease in hypertrophic cardiomyopathy-associated cardiac remodeling and myocardial dysfunction [41]. Moreover, RAN treatment decreased oxidative stress and alleviated diastolic dysfunction in rats fed a high-salt diet to develop hypertension [42].

Williams and co-workers demonstrated that RAN was efficient in lowering diastolic dysfunction in spontaneously hypertensive rats, and its mechanism of action was associated with suppression of the enhanced late sodium current in the SHR, resulting in decreased Ca^2+^ overload [43]. Le et al. proposed that RAN elevated adenosine concentrations in coronary veins in anaesthetized dogs, both at rest and during dobutamine-caused myocardial ischemia, mostly via enhancing the function of the cytosolic-5′-nucleotidase enzyme [33].

In individuals with CCS, RAN has been proposed as a way to increase myocardial perfusion and lessen mechanical compression of coronary microcirculation [44]. RAN enhanced coronary flow reserve in 58 patients with angina and myocardial ischemia but no obstructive coronary artery disease. This was likely because it improved abnormal coronary autoregulation, which decreased the baseline diastolic coronary flow rate and elevated the hyperemic diastolic coronary flow rate [45]. Furthermore, angina was found to improve when RAN was given in comparison to a placebo in a small trial involving women who had angina, signs of myocardial ischemia, but no obstructive coronary artery disease (CAD). There was also a trend towards improvement in the anomalies of myocardial perfusion detected by cardiac magnetic resonance imaging (CMR imaging). Additionally, compared to women with CFR >3.0, those with CFR ≤3.0 had a markedly increased myocardial perfusion reserve index (MPRI) while using RAN versus placebo [46]. RAN therapy also increases arginine plasma values and reduces oxidative stress in a randomized controlled study of 20 patients with unstable angina pectoris and acute cardiac ischemia [47].

Chou and colleagues discovered that RAN notably reduced action potential time, Cai transient time, and Cai decay duration, improved conduction inhomogeneity, and repressed arrhythmogenic alternans induction in db/db mouse hearts with acute IR damage [48]. Wolfes et al. studied the impact of RAN paired with various selective NCX-blockers in an isolated whole-heart model of AF in rabbits and discovered that both combinations extended aERP and aPRR and thereby reduced the development of AF [49]. In levosimendan-produced atrial fibrillation, RAN has a prominent antiarrhythmic effect, and the primary mechanism is a slight delay in repolarization and refractory period, which preserves the atrial myocardium against premature excitement and atrial fibrillation in rabbits [50]. Additionally, RAN appeared to have a dose-dependent antiarrhythmic impact on pacing-induced reentrant ventricular arrhythmias during the late phase of myocardial infarction in anaesthetized rabbits [51].

Markandeya et al. revealed that RAN inhibited late INa, which shortened APD and abolished triggered activity in Lmna (N195K/N195K) ventricular myocytes [52]. RAN has been found to enhance redox balance and mitochondrial activity in the atrium of rats suffering from acetylcholine-CaCl_2_-mediated atrial fibrillation [53]. RAN reduced delayed repolarization, aberrant electrical activity, and greater late sodium currents in elderly rats continuously exposed to low testosterone, all of which encouraged maladaptive electrical remodeling in ventricular myocytes [54].

Mustroph and colleagues investigated the beneficial effect of RAN on ethanol-induced atrial fibrillation and discovered that it efficiently suppressed atrial fibrillation by altering the activity of the CaMKII-dependent NaV1.5 channel [55]. RAN also inhibited electrical remodeling, causing atrial fibrillation in HL-1 atrial myocytes through modification of the PI3K/Akt signaling axis [56]. Opacic and his group emphasized that RAN effectively lengthened the atrial effective refractory time and lowered the atrial conduction rate at baseline and after 2 days of AF in a goat model of lone AF [57]. RAN was also compared with vernakalant for cardioversion of acutely produced AF in 15 rabbit hearts. AF was produced with atrial burst pacing and acetylcholine/isoproterenol. RAN besides vernakalant showed equal efficacy in preventing AF [58].

Similarly, recent research in horses found that, in comparison with single medications, the combination of dofetilide and RAN improved the antiarrhythmic effects on acutely generated AF, influencing cardioversion time, susceptibility at AF, and AF latency [59]. The combination of RAN and ivabradine has been evaluated in AF in pigs and the combined effect of these two drugs reduced ventricular rate via decreasing conduction at the AV node (increased A-H period) and minimizing the dominant AF frequency [60].

RAN was tested to assess its effects in a canine model of heart disease. It blocks atrial fibrillation in animals by lengthening the atrial refractory duration and atrial conduction time. No pro-arrhythmic influence was apparent on the ventricle [61]. Also, RAN administration avoided VT in the porcine model of catecholaminergic polymorphic ventricular tachycardia and decreased the T-wave length [62]. RAN has also been found to be non-inferior to lidocaine and sotalol in avoiding ischemia-reperfusion-induced ventricular tachycardia in a rat model [63]. Malavaki and team examined the vasorelaxant action of RAN and nicardipine on the rabbit aorta. Researchers found that RAN has a synergistic interaction with nicardipine to trigger vaso-relaxation in rabbit aortas [64]. RAN inhibited the occurrence and minimized the duration of action potentials in HL-1 cells, resulting in an antiarrhythmic response [65].

In another study, RAN reduced HOCl-LDL-associated alterations in cardiac contractility and electrophysiology, including arrhythmias in primary cardiomyocytes [66]. Del-Canto et al. found that RAN ameliorated the electrophysiological effects responsible for the stretch-induced modification of HL-1 cell fibrillatory activation patterns by altering the rise in activation rate and preserving the magnitude of activation [67]. RAN modified the ECG abnormalities, diminished Ca^2+^ sparks and abnormal waves, lowered the *in vitro* events and the frequency of arrhythmias noticed in isolated cardiomyocytes of hypothyroid mice [68]. Two preclinical studies of RAN demonstrate promise in preventing long QT syndrome in rats. RAN suppressed QT prolongation, prevented early after depolarizations, and reduced the duration of torsades de pointes [69, 70].

RAN showed antiarrhythmic efficacy against AT (Atrial Tachycardia) elicited by rapid burst stimulation in anaesthetized rabbits [71]. Nunoi and his team examined the anti-atrial fibrillatory effect and pharmacological safety characteristics of RAN in halothane-anesthetized dogs. Researchers found that RAN had little effect on ventricular early repolarization *in vivo*, but it did extend late repolarization with no danger of re-entrant arrhythmias [72].

Wolfes and colleagues evaluated the impact of RAN in combination with several specific NCX-blockers in an isolated whole-heart AF model. Both combinations increased the atrial effective refractory time while decreasing the frequency of AF episodes [49]. Aidonidis et al. investigated whether co-treatment of RAN-AMIO would show additive antiarrhythmic effects. RAN notably improved the propagation duration of fast atrial depolarizations and enhanced the AMIO-mediated mild elevations in aPRR [73]. Miranda and co-workers explored the influence of RAN on healthy cardiomyocytes as well as a cellular model of type 3 long QT syndromes (LQT3). RAN had a small effect on sarcomere shortening in healthy ENDO and EPI cells, and it reduced arrhythmias caused by INaL to the same rate as ENDO and EPI cells [74].

Eleclazine and RAN reduced the AF window and AF burden in association with the inhibition of both endogenous and enhanced atrial late INa with half maximal inhibitory concentrations (IC50) of 1.14 and 9.78 μM and 0.94 and 8.31 μM, respectively [75]. RAN normalized AV-conduction in Scn5a1798insD/+ mice by preventing the mutation-induced increase in intracellular sodium ([Na^+^]i) and calcium ([Ca^2+^]i) concentrations [76]. RAN also inhibited TASK-1 channels, and inhibition of TASK-1 may contribute to the observed antiarrhythmic effects of RAN [3]. RAN suppressed CaT alternans and decreased the Ca^2+^-voltage coupling gain in a dog HF model, reducing arrhythmogenic cardiac alternans [77]. RAN has continued to yield amazing outcomes, such as the cessation of acutely caused AF in horses via cardioversion [78]. RAN partially prevented action potential and QT interval prolongation in 4-week-old Scn5a^+/ΔQKP^ mice and suppressed arrhythmias [79].

Ke and colleagues examined how Ca^2+^ homeostasis was affected in CKD mice and discovered that RAN, by controlling CaMKII, PLB, and late Na^+^ current, reduced the length of the QT interval and the development of cardiac arrhythmogenesis [80]. Huang et al. investigated the role of FGF23 in activating the INa-Late, resulting in calcium imbalance and increasing PV arrhythmogenesis, and found that RAN-reduced FGF23 enhanced beating rates, calcium fluctuations, and mitochondrial ROS in PV cardiomyocytes [81].

In human atrial myocytes, RAN alone or when combined with low-dose dronedarone prolonged APD, increased cellular hyperpolarization, and decreased SR Ca (2+) leakage [82]. RAN has been observed to possess a similar effect to mexiletine in terms of action potential period shortening, with less paradoxical action potential duration prolongation in LQT3 mutant cells [83]. In the rabbit heart model, RAN perfusion substantially decreased the number of breakthrough-type excitations (BEs) in the ischemic border zone (BZ) and mitigated ischemia-caused shortening of action potentials in the BZ without influencing conduction velocity, most likely because of IKr repression [84]. RAN also decreased VT load and implanted cardioverter-defibrillator (ICD) shocks in 11/12 individuals receiving drug-refractory shocks [85]. RAN also proved to be effective, well-tolerated, and safe in reducing ventricular arrhythmia episodes and ICD interventions in patients with recurrent antiarrhythmic drug-refractory events [86]. After analyzing a group of AF patients on RAN, Black-Maier et al. discovered that the medication is linked to decreased AF DF but not altered organization index or fibrillatory wave amplitude [87]. RAN, a late I (Na) blocker, appeared to possess antiarrhythmic effects, according to continuous ECG monitoring of patients admitted for acute coronary syndrome within the first week [88].

RAN was tested in patients having coronary artery disorder and paroxysmal AF who used to have a double chamber pacemaker able to detect AF. RAN 375 mg twice each day compared with placebo shortened average AF duration and mean AF length. There was no substantial variation in QTc. The 500 mg and 750 mg arms combined showed a reduction in AF recurrence with borderline statistical significance [89]. RAN has also been demonstrated to result in a greater conversion rate of AF to normal sinus rhythm when administered in combination with amiodarone than amiodarone alone in randomized clinical research including 121 patients [90]. Tsanaxidis et al. found that a single 1000 mg daily treatment of RAN when given with amiodarone leads to a faster recovery to sinus rhythm and a better sinus conversion rate than amiodarone alone. The addition of RAN had no detrimental effect on left ventricular activity [91]. The additive value of RAN to amiodarone in AF has been confirmed by two meta-analyses. The use of RAN accelerates the time for AF cardioversion. It also helps avoid new-onset AF in people with disabilities rhythm of sinus [92, 93].

The HARMONY study demonstrated that combining moderate dosages of oral RAN with decreased doses of dronedarone effectively ameliorated the AF burden in individuals with paroxysmal AF and was tolerated satisfactorily [94]. Many other small trials have found that RAN decreases conversion time from atrial fibrillation to sinus rhythm. It also increases heart function following coronary artery bypass grafting (CABG) [95–98]. Another clinical study explored the impact of RAN on AF in postoperative atrial fibrillation (POAF). Patients having heart valve and/or heart bypass surgery have been involved. The addition of RAN to normal treatment markedly decreased the frequency of POAF. There was no effect on the stay in the intensive care facility or cardiovascular death, but the rate of cardiovascular readmission decreased by 30 days [99].

**FIGURE 2:**
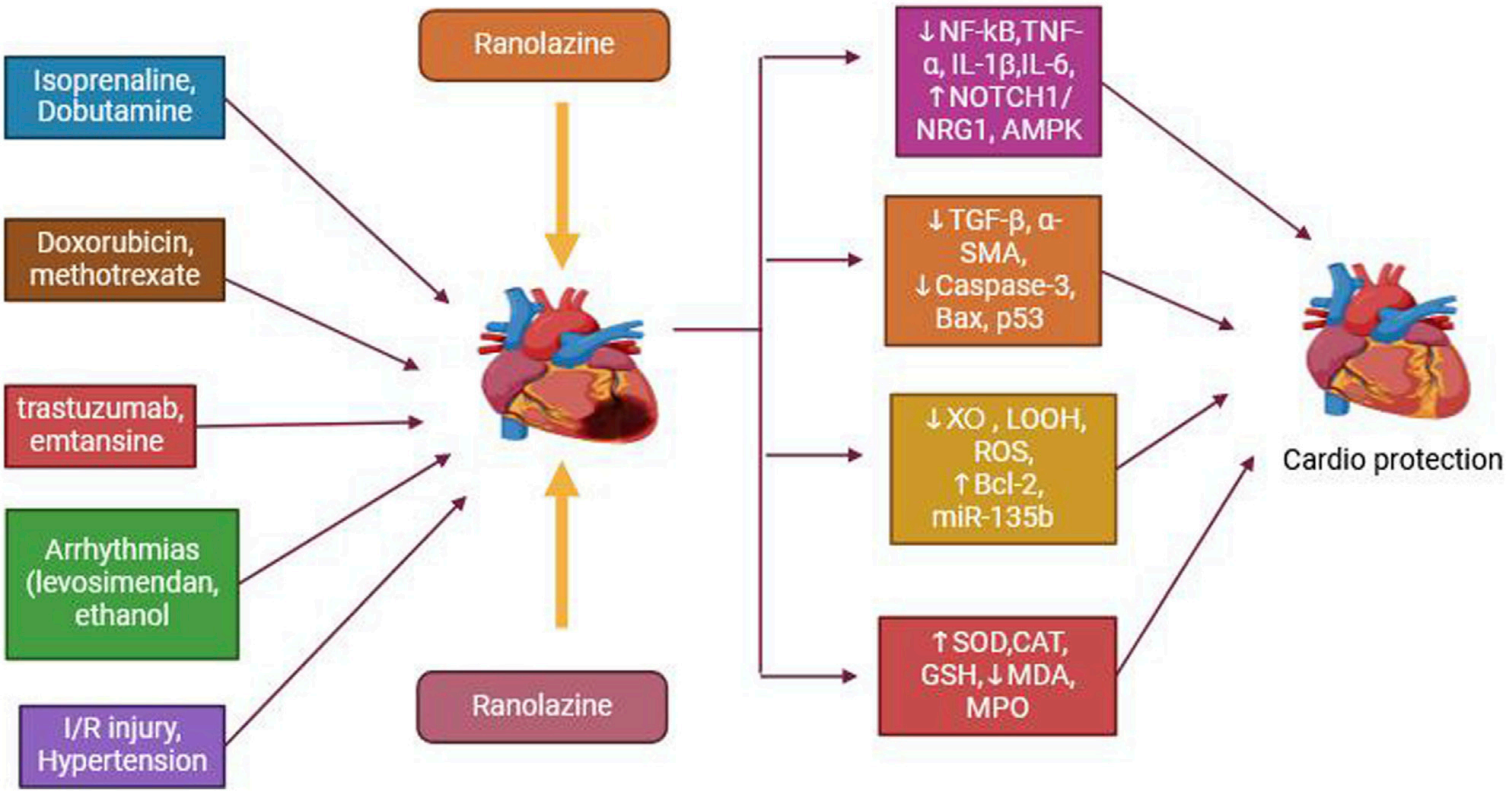
Therapeutic efficacy of ranolazine in heart dysfunction.

In patients experiencing acute coronary syndrome without ST-segment acceleration, RAN has been found to minimize the rate of non-sustained ventricular tachycardias and atrial fibrillation (AF) [100]. In another small study of eight patients with long QT syndrome type 3 (LQTS3), RAN was demonstrated to successfully decrease the QT period, hence reducing the frequency of ventricular arrhythmias [101]. Figure 2 shows the therapeutic efficacy of RAN in heart dysfunction. RAN inhibits TNF-α, IL-1β, NF-κB, Caspase-3, Bax, ROS, and Ca^2+^ levels and activates Notch, AMPK, and miR-135b Bcl-2, resulting in improved outcomes for cardiac arrhythmia, cardiac fibrosis, cardiac injury, and myocardial infarction.

Cempaka Putri et al. [102] conducted a systematic review and meta-analysis on the efficacy of using RAN to improve diastolic performance and exercise capacity in heart failure with preserved ejection fraction. It was established that RAN was significantly efficacious in improving diastolic performance in heart failure patients with preserved ejection fraction, with no significant effect on blood pressure, heart rate, and ventricular repolarization rate (shortening of the QT interval).

## Neuroprotective effects

Piano and colleagues investigated the protective effect of RAN on microglia cells stimulated by LPS and found that RAN counteracts the neurotoxic effect of LPS-activated microglia on 661W neuronal cells [103]. RAN dramatically enhanced cell survival and growth in cultured astrocytes at any tested dose while decreasing LDH loss, Smac/Diablo activity, and Caspase-3 action, demonstrating a decreased rate of cell death [104].

Akgul and co-workers examined the beneficial effect of RAN in a brain I/R model of rats and concluded that RAN helped in cerebral recovery by increasing Bcl-2 and NA levels and decreasing AChE, TNF-α, and ACP levels [105]. Kahlig and team studied the antiepileptic action of RAN in hippocampus neurons and discovered that at therapeutic doses, RAN lowered the action potential firing rate of hippocampal neurons in response to recurrent depolarizing current injections by stabilizing the inactivated states of Na^+^ channels [106]. Peters et al. investigated the possibility of RAN as an anticonvulsant and found that RAN affected Nav1.2 channels, lowering macroscopic currents and slowing the recovery of rapid and slow inactivation of the Nav1.2 channel in hamster ovary cells stably expressing the rat Nav1.2 channel [107].

**FIGURE 3:**
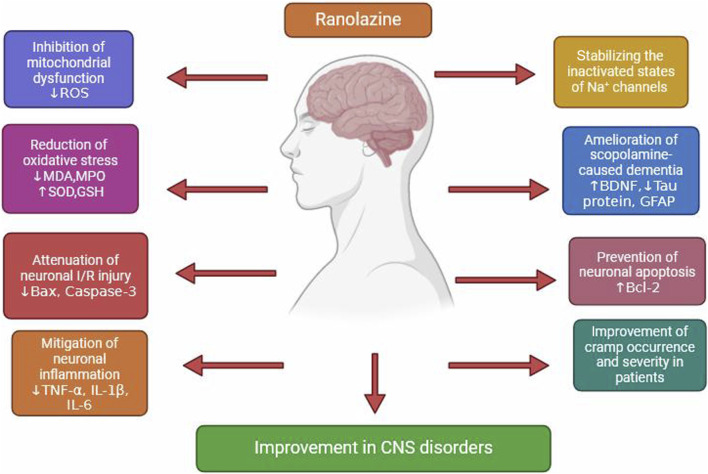
Promising therapeutic applications of ranolazine in neuronal injury.

In a rat model of DOX-induced neurotoxicity, RAN reduced brain inflammation, improved BBB integrity, alleviated brain mitochondrial dysfunction, inhibited apoptosis, and preserved microglial structure and hippocampal plasticity [108]. Samir et al. revealed that RAN has a unique neuroprotective function against scopolamine-caused dementia in rats via antioxidative, anti-inflammatory, and anti-apoptotic actions as well as regulation of GFAP, BDNF, and Tau protein levels [109]. In diabetic neuropathy rats, RAN and pioglitazone have separately altered evoked-pain activity, lowered sciatic TNF-α and 1L-1β levels, decreased levels of Nav1.7 channels, and enhanced expression of the spinal PPAR-γ gene [110]. Chandrashekhar and colleagues conducted an open-label dose-ascending trial of RAN in 14 people with amyotrophic lateral sclerosis, examining muscular cramp symptoms. It was discovered that RAN improved cramp occurrence and severity, which supports its study into muscular cramps [111]. Figure 3 shows promising therapeutic applications of RAN in neuronal injury.

RAN primarily activates anti-apoptotic and neuronal survival pathways such as Bcl-2. It also suppresses Caspase-3, TNF-α, IL-1β, IL-6, ROS, and other factors that promote neuronal death.

## Renal protective effects

RAN substantially reduces renal ischemia-reperfusion damage in rats, which was accomplished by modulating the inflammatory reactions via a noteworthy drop in renal tissue level of HMG box1, IL-1ß, downregulation of the Notch2/Hes1 signaling pathway, and anti-oxidant action [112]. According to Abbas and teammates, RAN dramatically reduces renal ischemia-reperfusion damage in rats by increasing Bcl2 protein levels, decreasing Bax and TNF-alpha levels, and inhibiting the oxidative stress biomarker F2-isoprostane and Notch2/Hes1 signaling cascade [113]. Nayaka and Vaish revealed that RAN therapy dramatically lowered blood glucose levels, preserved renal functions, and maintained near-normal renal structure due to its glycemic management and anti-inflammatory and anti-oxidative effects against STZ-caused diabetic nephropathy in rats [114]. Ma and associates investigated the protective effect of RAN in contrast-induced acute renal injury (CI-ARI). Pre-treatment of RAN in CI-ARI mice showed no effect on total blood pressure but significantly enhanced renal perfusion, decreased contrast-associated microcirculation disruption, accelerated renal capillary thickness, and ameliorated renal vascular permeation [115]. Yusuf et al. investigated administering RAN as a preventative for patients with low renal failure having PCI and discovered that it might prevent the development of CIN [116].

## Pain and inflammation

RAN inhibited DRG neuron hyperexcitability by interfering with inactivated Na (+) channels, and these activities could lead to its anti-allodynic action in animal models of neuropathic pain [117]. Furthermore, at a dose routinely employed in clinical settings, RAN was discovered to be efficacious in preventing the fast firing of DRG neurons with WT Nav1.7 channels, reducing neuropathic and inflammatory pain [118].

RAN has been demonstrated to attenuate pain behavior in animal models of acquired neurotic pain; however, the drug’s effects on cold-induced pain were more potent than mechanical allodynia, and the reduction in pain was only temporary, lasting only 30–90 min based on oral or *i.p* delivery [119]. Casey et al. assessed the analgesic efficacy of RAN in complete Freund’s adjuvant-mediated inflammatory pain in rats. They found that RAN exhibited a dose-dependent analgesic effect [120]. According to Gould et al. RAN at 30 mg/kg efficiently ameliorated the painful mechanical allodynia related to demyelination injury, which was induced by the administration of doxorubicin [121].

Naveena and colleagues investigated the anti-inflammatory efficacy of RAN in acute and sub-acute inflammation models in rats and found that RAN substantially lowered paw oedema volume and histological sections revealed a reduction in granulation tissue development [122]. Lenz and coworkers claimed that Na^+^ suppression by RAN resulted in lower expression of adhesion molecules and pro-inflammatory cytokines as well as reduced adherence of leukocytes to activated endothelium *in vitro* and *in vivo* [123].

## Antidiabetic activity

Jordá et al. found that RAN improved insulin consequences in primary culture astrocytes by increasing anti-inflammatory facilitators like PPAR-γ, decreasing pro-inflammatory agents like COX-2, and boosting the action of Mn-SOD and components of the AKT-eNOS and ERK signaling cascade [124]. Bashir and colleagues investigated the antidiabetic efficacy of RAN against STZ-caused diabetes in rats. It was observed that RAN improved plasma fasting glucose levels and also exhibited a positive effect on the lipid profile [125].

Non-clinical investigations showed that RAN reduced fasting and non-fasting glucose levels and preserved pancreatic β-cells in STZ-induced diabetic mice [126]. In animal models of diabetes, RAN lowered postprandial and basal glucagon concentrations, resulting in a drop in hyperglycemia, demonstrating that RAN’s glucose-lowering actions might be achieved via the blocking of sodium channels in pancreatic alpha cells [127]. Guerra-Ojeda explored the potential beneficial effects of RN on insulin activity in the rabbit aorta. They discovered that RAN improved vascular sensitivity to insulin, reducing tissue resistance to the hormone by raising the activities of p-eNOS/eNOS and pAKT/AKT [128]. Cassano et al. assessed the effects of RAN on glucose metabolism and cognitive performance in a T2DM model of Wistar rats and concluded that RAN improved glucose metabolism, enhanced learning and long-term memory, and modified the pro-inflammatory characteristics of diabetic mice [129]. Another study revealed the protective impact of RAN on hippocampal neurodegeneration and astrocyte activation in an STZ T2DM rat model and found that RAN reduced T2DM-induced neuronal injury and loss [130].

A post-hoc examination of the MERLIN-TIMI 36 trials indicated a 0.64 percent drop in HbA1c in diabetic patients who took RAN relative to those who did not. Fasting plasma glucose was also notably decreased by an average of 25.7 mg/dL [131]. Pettus et al. recently verified the MERLIN-TIMI 36 trial results. They investigated the use of high-dose RAN for glycemic control in addition to glimepiride background treatment (4 mg/day) in type 2 diabetes patients with an average baseline HbA1c level of 8.1% [132].

The CARISA research showed that RAN is effective at reducing HbA1c levels in patients with unstable angina. In this assay, HbA1c was not a given result, and further stratification of results based on insulin or oral antihyperglycemic use was not possible [133]. A later randomized analysis of 465 T2D patients with an average HbA1c∼8 controlled by lifestyle alone at the start indicated that RAN resulted in higher declines in HbA1c than placebo at 24 weeks (mean difference = 0.56, p < 0.0001) [134]. In addition to its anti-ischemic and antianginal properties, RAN demonstrated the capacity to reduce HbA1c in individuals with coronary artery disease and T2DM in two clinical investigations [135]. In a group of patients with T2D and CCS, RAN, in addition to usual anti-ischemic and glucose-lowering medication, also showed effectiveness in restoring endothelial function and glycemic status, as measured by Hb1Ac and short-term GV indices [136].

## Muscle disorder

When 10 µM RAN was applied for treating C2C12 myoblasts throughout cell growth, transformation, and the development of new myotubes, it increased the levels of myogenic regulator factors (Myf5 and MyoD), suppressed cell progression factor, decreased ROS, and preserved mitochondrial homeostasis [137]. Tomczyk and colleagues evaluated the positive effects of RAN on skeletal muscle function and metabolism in dyslipidemic rats. They learned that RAN-mediated suppression of FFA oxidation in ApoE/LDLR −/− mice resulted in reduced exercise performance and total adenine nucleotide pool [138].

Torcinaro et al. explored the efficacy of RAN in preventing skeletal muscle dysfunctions associated with aging and discovered that RAN administration dramatically enhanced the muscular strength of elderly mice via up-regulating antioxidant and mitochondrial genes, and by increasing NADH-dehydrogenase function [139]. Novak and collaborators revealed that RAN improved muscle functioning compared to mexiletine without major side effects in a mouse model of myotonia congenita [140]. An open treatment study with RAN at a dose of 2 × 500 mg in 13 patients with chloride channel myotonia showed a significantly reduced EMG myotonia, and according to patient reports, significantly reduced muscle stiffness, and, to a lesser extent, a reduction in muscle weakness and reduced myotonia in clinical tests [141].

Lorusso et al. recently investigated the efficacy of RAN in an open-label trial of 10 patients having paramyotonia congenita and concluded that RAN dramatically reduced both subjective symptoms and clinical myotonia [142]. A phase 2 study is underway to assess the efficacy of RAN in MC, paramyotonia congenita, and Type 1 myotonic dystrophy. Patients with the above conditions were randomized to receive RAN 500 mg twice daily for 2 weeks followed by 1000 mg two times daily for 2 more weeks, compared to placebo. Primary outcomes are quality of life measurements for health and neuromuscular disease, and EMG to assess for changes in muscle potentials and performance. It is a phase 2 trial to mainly assess the safety profile of the drug in these neuromuscular conditions (NCT02251457).

## Pulmonary hypertension

Lee and colleagues investigated the preventive function of RAN against monocrotaline-caused PAH in rat models and found that RAN attenuated ventricular hypertrophy, B-type natriuretic peptide values, fibrosis activation, and cardiovascular mortality [143]. Rocchetti et al. have established that RAN inhibited constitutive elevation of the late sodium current, thereby delaying the development of myocardial remodeling in an experimental rat model of PAH induced by monocrotaline [144]. Teixeira-Fonseca et al. proved that RAN attenuated right ventricular hypertrophy while improving P wavelength and QT period in a monocrotaline-caused PH rat model [145]. In an *in vivo* study, acute treatment of RAN dramatically decreased isoproterenol-caused ventricular tachycardia/ventricular fibrillation and related cardiovascular mortality in rats with pre-existing pulmonary arterial hypertension (PAH) and heart remodeling [146]. Furthermore, a pilot experiment at a single center revealed that 8 of the 11 recruited patients completed all the research exams. The WHO FC, RV function, and exercise tolerance findings revealed improvement without any changes to the invasive hemodynamic measures, and the RV size in PAH patients was decreased after 3 months of RAN medication [147].

A recent double-blind, randomized, placebo-controlled RAN trial (n = 9 RAN, n = 6 placebo) revealed that RAN therapy enhanced RV ejection fraction but not 6-min wall distance (6MWD), N-terminal pro-brain natriuretic peptide, or quality-of-life expectancy measures in patients having precapillary pulmonary hypertension [148]. Finch and colleagues observed that the approved antianginal drug RAN improved cardiopulmonary hemodynamics, functional status, and exercise tolerance in both short-term and long-term (average time on drug approximately 2 years) plans in a cohort of patients with PH-HFpEF [149]. A Phase Ib investigation including 12 PAH patients showed no statistical significance in terms of adverse events between the control and RAN groups after a 12-week follow-up period. This outcome demonstrated the safety of the RAN therapy but did not accomplish the therapeutic aim, partly because the study medication did not reach a therapeutic serum level [150].

## Peripheral arterial disease

An animal model demonstrated that injecting RAN into the femoral artery causes a long-lasting dilatation of the artery, equivalent to that produced by nitroglycerin. This outcome might be attributed to α1-adrenergic receptor inhibition, which does not affect heart rate and systemic blood pressure [151]. In a pilot research study including 45 patients with irregular claudication, RAN 1000 mg BID elicited an improvement in peak walking time in comparison with placebo. Though RAN did not ameliorate the ankle-brachial index at rest, patients with extremely irregular claudication had approximately 40 percent improvement in walking time relative to placebo compared to cilostazol [152].

## Hepatoprotective effects

Saed and his colleagues assessed the efficacy of RAN in attenuating obesity-induced NAFLD and hyperglycemia and concluded that RAN therapy enhanced glucose tolerance and lowered hepatic triacylglycerol levels in obese mice through increasing the activity of mRNA, which plays a role in modulating lipogenesis [153]. Al Batran stated that in a mouse model of nonalcoholic fatty liver disease, RAN significantly improved glucose oxidation via increasing PDH function [154]. Pzolat and colleagues investigated the preventive effects of RAN against MTX-induced liver injury in rats and found that RAN could attenuate MTX toxicity by reducing MDA and MPO values, enhancing SOD, CAT, and GSH levels, and improving mononuclear inflammation, vascular congestion, and fibrosis [155].

## Testicular injury

Bilge et al. evaluated the protective effect of RAN in a testis torsion rat model induced by I/R and demonstrated that RAN protected against testicular damage by reducing MDA levels and improving histopathological scores [156].

## Other activities

A recent study revealed that prolonged RAN treatment enhanced energy metabolism by enhancing muscle ATP content and slowing muscular strength reduction in a mouse model of amyotrophic lateral sclerosis (ALS) [157]. Marchio et al. studied the impact of RAN on vascular function and adrenergic response in human saphenous veins. They observed that RAN reduced adrenergic vasoconstriction by acting as an α_1_ antagonist and enhancing the huge conductance Ca^2+^activated K^+^ channel [158].

## Molecular mechanisms of non-cardiac effects of Ranolazine

The non-cardiac effects of RAN have been associated with various molecular mechanisms. These include ion channel (late sodium and calcium) modulation, adrenergic receptor antagonism, and metabolic effects, which collectively result in improved cellular ion homeostasis, reduced oxidative stress, and mild vasodilation in non-cardiac tissues [159]. RAN selectively inhibits the late phase of the inward sodium current and elicits a mild blocking effect on L-type calcium channels. The impacts of these effects include a reduction in intracellular sodium, consequent decrease in calcium overload via the sodium–calcium exchanger (stabilization of cellular ion homeostasis and reduction of cellular stress in tissues), and weak vasodilatory properties with consequences on vascular smooth muscle tone and peripheral circulation [159, 160]. RAN elicits antagonistic action at alpha-1 and beta-1 adrenergic receptors present in vascular, nervous, and other tissues. This antagonistic action contributes to the modulation of vascular tone and sympathetic nervous system effects, devoid of significant changes in heart rate or blood pressure [160].

RAN invokes inhibition of delayed rectifier potassium current, which, beyond cardiac tissue, could influence electrophysiological properties in other excitable tissues [160]. RAN partially inhibits fatty acid oxidation at higher concentrations, leading to alteration of metabolic processes in non-cardiac tissues; this may lead to improvement of cellular energy efficiency under stress conditions [161].

## Conclusion

RAN is a well-known selective INa,L inhibitor and the most commonly utilized antianginal agent. This amazing substance is mostly used to treat chronic angina (chest pain). RAN is an add-on medicine for the relief of symptoms of individuals suffering from stable angina pectoris and those who are poorly controlled or intolerant to first-line antianginal therapy. However, an exciting surge of interest is rising around the possibility of RAN being repurposed for a varied array of health conditions. This review article investigates RAN’s varied pharmacological actions, shedding light on its prospective possibilities outside the field of antianginal drugs. The review demonstrates its promise in treating an astounding variety of illnesses, from anticancer activity and neuroprotection to renal and liver protection, renal antidiabetic advantages, and anti-inflammatory capabilities.

The repurposing of RAN offers clinical promise in various health conditions, including pulmonary hypertension, arrhythmia, heart failure, metabolic disease, and oncology, in view of its unique ion channel modulation, metabolic effects, and anti-inflammatory properties. These benefits of RAN, coupled with its safety profile, offer translational opportunities for diverse therapeutic benefits.

## Future perspective

RAN exhibits pleiotropic properties, demonstrating several mechanisms of action and protective benefits against various disease models already established. Given that inflammation and oxidative stress are the fundamental contributors to almost all human diseases, medications that might impede these processes are expected to be beneficial in various medical conditions. The review focuses on the many pharmacological properties of RAN, as it has been demonstrated to produce these effects.
